# Structurally Diverse Metal Coordination Compounds, Bearing Imidodiphosphinate and Diphosphinoamine Ligands, as Potential Inhibitors of the Platelet Activating Factor

**DOI:** 10.1155/2010/731202

**Published:** 2010-06-28

**Authors:** Alexandros B. Tsoupras, Maria Roulia, Eleftherios Ferentinos, Ioannis Stamatopoulos, Constantinos A. Demopoulos, Panayotis Kyritsis

**Affiliations:** ^1^Biochemistry Laboratory, Faculty of Chemistry, National and Kapodistrian University of Athens, Panepistimiopolis, Zografou, 15771 Athens, Greece; ^2^Inorganic Chemistry Laboratory, Faculty of Chemistry, National and Kapodistrian University of Athens, Panepistimiopolis, Zografou, 15771 Athens, Greece

## Abstract

Metal complexes bearing dichalcogenated imidodiphosphinate [R_2_P(E)NP(E)R_2_′]^−^ ligands (E = O, S, Se, Te), which act as (E,E) chelates, exhibit a remarkable variety of three-dimensional structures. A series of such complexes, namely, square-planar [Cu{(OPPh_2_)(OPPh_2_)N-*O*, *O*}_2_], tetrahedral [Zn{(EPPh_2_)(EPPh_2_)N-*E*,*E*}_2_], E = O, S, and octahedral [Ga{(OPPh_2_)(OPPh_2_)N-*O*,*O*}_3_], were tested as potential inhibitors of either the platelet activating factor (PAF)- or thrombin-induced aggregation in both washed rabbit platelets and rabbit platelet rich plasma. For comparison, square-planar [Ni{(Ph_2_P)_2_N-*S*-CHMePh-*P*, *P*}X_2_], X = Cl, Br, the corresponding metal salts of all complexes and the (OPPh_2_)(OPPh_2_)NH ligand were also investigated. Ga(O,O)_3_ showed the highest anti-PAF activity but did not inhibit the thrombin-related pathway, whereas Zn(S,S)_2_, with also a significant PAF inhibitory effect, exhibited the highest thrombin-related inhibition. Zn(O,O)_2_ and Cu(O,O)_2_ inhibited moderately both PAF and thrombin, being more effective towards PAF. This work shows that the PAF-inhibitory action depends on the structure of the complexes studied, with the bulkier Ga(O,O)_3_ being the most efficient and selective inhibitor.

## 1. Introduction

Extensive research work over the last few years has revealed a remarkable structural variability of transition metal compounds bearing dichalcogenated imidodiphosphinate type of ligands, that is, [R_2_P(E)NP(E)R_2_′]^−^, E = O, S, Se, Te; R, R′ = various aryl or alkyl groups. These ligands have been shown to display great coordinating versatility, producing both single and multinuclear metal complexes, with a variety of bonding modes [1–3]. The coordinating flexibility of these (E,E) chelating ligands is attributed, mainly, to their large (*c* *a*. 4 Å) E⋯E bite, which would accommodate a range of coordination sphere geometries. For instance, it was recently shown that the [^i^Pr_2_P(Se)NP(Se)^i^Pr_2_]^−^  ligand affords both tetrahedral and square-planar complexes of Ni(II) [4], in agreement with an earlier observation on the analogous [Ph_2_P(S)NP(S)Ph_2_]^−^ ligand [5]. Moreover, the nature of the R and R′ peripheral groups of the [R_2_P(S)NP(S)R_2_′]^−^ ligand has been shown to affect the geometry of the complexes formed upon its coordination to Ni(II) [6, 7]. In a more general sense, depending on the nature of the metal ion, the chalcogen E atom and the R peripheral group, complexes bearing the above type of ligands were shown to contain rather diverse coordination spheres [8]. Such structural differences are of significant importance, as they are expected to lead not only to different stereochemical characteristics, but also to varied electronic properties of the metal site, which, in turn, could potentially result in significant biological reactivity [9].

The aim of this work was to investigate a series of structurally diverse metal coordination compounds bearing dichalcogenated imidodiphospinate ligands, as potential inhibitors of PAF (1-*O*-alkyl-2-acetyl-*sn*-glycero-3-phosphocholine). PAF is a phospholipid signalling molecule of the immune system and a significant mediator of inflammation. PAF transmits outside-in signals to intracellular transduction systems in a variety of cell types, including key cells of the innate immune and haemostatic systems, such as neutrophiles, monocytes, and platelets [10, 11]. In addition, it exhibits biological activity through specific membrane PAF-receptors, coupled with G-proteins. The binding of PAF on its receptor induces intracellular signaling pathways that lead to several cellular activation mechanisms, depending on the cell or tissue type [12]. It is well established that increased PAF-levels in blood or tissues lead to various inflammatory manifestations [10–12] such as cardiovascular, renal and periodontal diseases [13–16], allergy [17], diabetes [18], cancer [19] and AIDS [20].

A great variety of chemical compounds, both natural and synthetic, have demonstrated an inhibitory effect towards the PAF-induced biological activities, acting either through direct antagonistic/competitive effects by binding to the PAF-receptor, or through indirect mechanisms. In the latter case, the biofunctionality seems to correlate with changes in the membrane microenvironment of the PAF-receptor. Natural and synthetic PAF antagonists exhibit variable chemical structures that might lead to different pharmacological profiles. Since PAF is assumed to play a central role in many diseases, the effects of its antagonists have been widely studied in experimentally induced pathologies and in clinical studies [21–24].

The use of metal complexes as potential pharmaceutics is increasingly gaining ground [25, 26]. Particularly, complexes of Ga(III) bearing (O,O) chelating ligands have been studied thoroughly as promising nonplatinum compounds with superior anticancer activity and lower side effects [27, 28]. These complexes have also shown anti-inflammatory activity towards reumatoidis arthritis or Alzheimer's disease [25]. Cu(II) complexes have been investigated as anticancer agents, based on the assumption that endogenous metals may be less toxic [29]. In addition, Ni(II) and Cu(II) complexes have been screened for their *in vitro* antibacterial and antifungal activity [30], whereas Zn(II) complexes have been investigated as agents against diabetes mellitus and ulcer [31]. A mechanistic understanding of how metal complexes exhibit their biological activity is crucial to their clinical success, as well as to the rational design of new compounds with improved pharmacological properties. In that respect, the *in vitro* investigation of novel metal complexes with targeted biomolecules may prove extremely valuable, before the *in vivo* tests in animal models. 

In this study, we examined the *in vitro* effects of representative bis- or tris-chelated complexes of dichalcogenated imidodiphosphinate ligands, involving Cu(II), Zn(II) and Ga(III) centers, against PAF-induced biological activities. For this purpose, the potent inhibitory effect of these metal complexes was studied on PAF-induced platelet aggregation towards both washed rabbit platelets (WRPs) and rabbit platelet rich plasma (PRP). The complexes investigated contain diverse metal coordination spheres, exhibiting square-planar, tetrahedral and octahedral geometries. In addition, two square-planar complexes of Ni(II), bearing one bidentate diphosphinoamine ligand [32] and two halide ions were also investigated, with a view of revealing the necessary structural features, among this set of coordination compounds, that would ensure efficient and selective inhibition of PAF. Moreover, the inhibitory action of some of these complexes towards thrombin was also investigated, in order to probe their selectivity with respect to either the PAF- or the thrombin-dependent platelet aggregation.

## 2. Experimental Part

### 2.1. Materials and Methods

The following complexes were prepared according to published procedures: [Cu{(OPPh_2_)(OPPh_2_)N-*O*, *O*}_2_] [33], [Zn{(OPPh_2_)(OPPh_2_)N-*O*, *O*}_2_] [34], [Zn{(SPPh_2_)(SPPh_2_)N-*S*, *S*}_2_] [35], [Ga{(OPPh_2_)(OPPh_2_)N-*O*, *O*}_3_] [36], [Ni{(Ph_2_P)_2_N-*S*-CHMePh-*P*, *P*}Cl_2_] [37]. These complexes are abbreviated as Cu(O,O)_2_, Zn(O,O)_2_, Zn(S,S)_2_, Ga(O,O)_3_ and Ni(P,P)Cl_2_, respectively. The synthesis of the analogous to Ni(P,P)Cl_2_, bromide-containing Ni(P,P)Br_2_ complex, was carried out according to the published procedure [37], with the exception of using Ni(DME)Br_2_ (DME = 1,2-dimethoxyethane) as a starting material. The detailed synthesis and characterization of Ni(P,P)Br_2_ will be described elsewhere. The (OPPh_2_)(OPPh_2_)NH ligand was prepared as described in the literature [38]. The following metal salts, CuCl_2_, ZnCl_2_, Ga(NO_3_)_3_·9H_2_O and NiCl_2_·6H_2_O, were also tested for comparison purposes. All chemical reagents used were purchased from Sigma-Aldrich (St. Louis, Mo, USA).

UV-vis spectra were recorded in a Varian Cary 3E spectrophotometer. 

Bovine serum albumin (BSA), PAF (1-*O*-hexadecyl-2-acetyl-*sn*-glycero-3-phosphocholine), thrombin and analytical solvents for the biological assays were purchased from Sigma.

Centrifugations were performed in a Heraeus Labofug 400R and a Sorvall RC-5B refrigerated super-speed centrifuge (Sigma-Aldrich). Aggregation studies were performed in a Chrono-Log aggregometer (model 400, Havertown, Pa, USA) coupled to a Chrono-Log recorder (Havertown) at 37°C with constant stirring at 1200 rpm. 

### 2.2. Biological Assay on WRPs and Rabbit PRP

The potential inhibitory effect of a range of metal complexes towards PAF-related biological activities was estimated by biological assays based on WRPs aggregation [10]. The examined metal complexes were first dissolved in dimethylsulfoxide (DMSO), at an initial concentration of (4–8) × 10^−3^ M. Subsequently, different aliquots of the complexes' solutions were added in a BSA solution (2.5 mg BSA/mL of saline). PAF was also dissolved in the same BSA solution. The metal salts were dissolved in saline. The platelet aggregation induced by PAF (4.4 × 10^−11^ M final concentration in the aggregometer cuvette) or by thrombin (0.1 IU) on WRPs, was measured before (considered as 0% inhibition) and after the addition of the sample examined. A linear plot of inhibition percentage (ranging from 20% to 80%) versus the concentration of the sample was established for each metal complex. From this curve, the concentration of the sample that inhibited 50% of the PAF-induced aggregation (IC_50_) was calculated. Biological assays were performed several times (*n* > 3), according to methods of Demopoulos et al. [10] and Lazanas et al. [39], so as to ensure reproducibility. The same procedure was also followed in the case of rabbit PRP, as previously described [40].

### 2.3. Statistical Methods

All results were expressed as mean ± standard deviation (SD). The t-test was employed to assess differences among the IC_50_ values of each metal complex against either the PAF- or thrombin-induced aggregation. Differences were considered to be statistically significant when the statistical p value was smaller than 0.05. Data were analyzed using a statistical software package (SPSS for Windows, 16.0, 2007, SPSS Inc. Chicago, IL) and Microsoft Excel 2007.

## 3. Results

### 3.1. Molecular Structures and Stability of the Complexes

**Figure 1:**
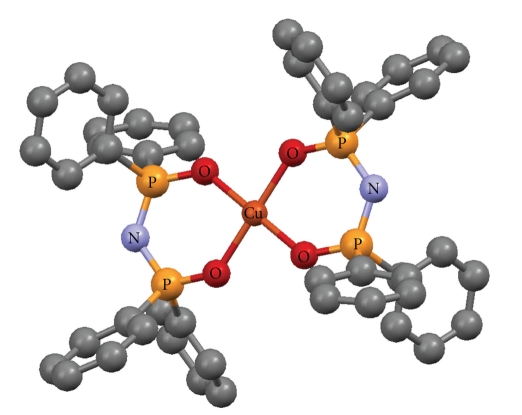
Crystal structure of [Cu{(OPPh_2_)(OPPh_2_)N-*O*, *O*}_2_] [33]. Color code: Cu (brown), O (red), P (orange), N (light blue), C (grey).

**Figure 2:**
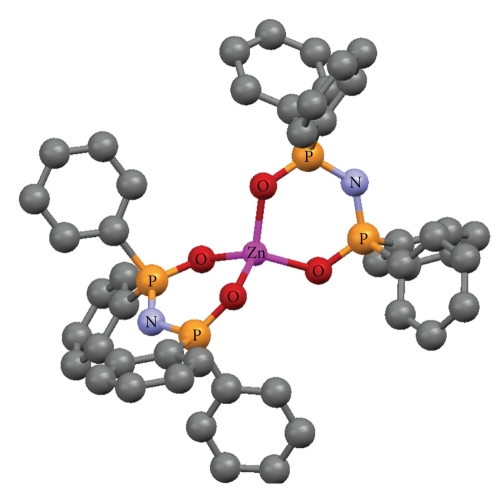
Crystal structure of [Zn{(OPPh_2_)(OPPh_2_)N-*O*, *O*}_2_] [34]. Color code: Zn (pink), O (red), P (orange), N (light blue), C (grey).

**Figure 3:**
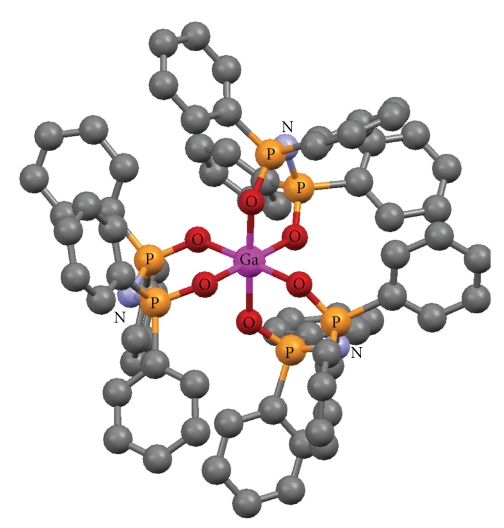
Crystal structure of [Ga{(OPPh_2_)(OPPh_2_)N-*O*, *O*}_3_] [36]. Color code: Ga (pink), O (red), P (orange), N (light blue), C (grey).

**Figure 4:**
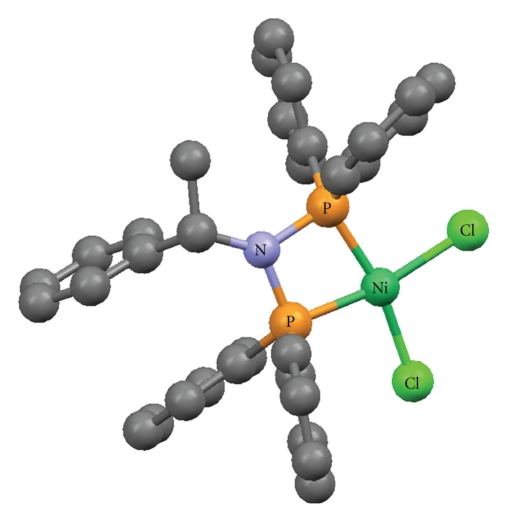
Crystal structure of [Ni{(Ph_2_P)_2_N-*S*-CHMePh-*P*, *P*}Cl_2_] [37]. Color code: Ni (green), Cl (light green), P (orange), N (light blue), C (grey).

**Figure 5:**
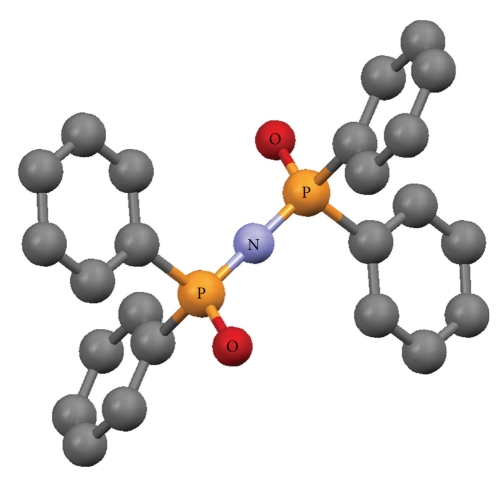
Crystal structure of the (OPPh_2_)(OPPh_2_)NH ligand [41]. Color code: O (red), P (orange), N (light blue), C (grey).

The crystallographic structures of Cu(O,O)_2_ [33], Zn(O,O)_2_ [34], Ga(O,O)_3_ [36] and Ni(P,P)Cl_2_ [37], as well as the (OPPh_2_)(OPPh_2_)NH ligand [41] have been already described (Figures 1–5). A variety of metal core geometries is demonstrated: Cu(O,O)_2_ and Ni(P,P)Cl_2_ are square-planar, whereas Zn(O,O)_2_ is tetrahedral and Ga(O,O)_3_ is octahedral. The Zn(S,S)_2_ and Ni(P,P)Br_2_ complexes are expected to be structurally similar to Zn(O,O)_2_ and Ni(P,P)Cl_2_, respectively. UV-vis absorption spectra of the light blue DMSO solutions of Cu(O,O)_2_ confirmed that the complex was stable for the time-span of the study. This is expected since Cu(O,O)_2_ and the rest of the dichalcogenated imidodiphosphinate complexes, contain highly stable six-membered M-E-P-N-P-E chelating rings [1, 2]. On the other hand, for Ni(P,P)_2_X_2_, X = Cl, Br, the intensity of the absorption maximum was gradually decreasing. Therefore, degradation of the complexes at some extent is likely, which is expected to affect their inhibitory action.

### 3.2. Inhibitory Effect of Metal Complexes and Metal Salts towards the PAF-Induced WRP's Aggregation

**Figure 6:**
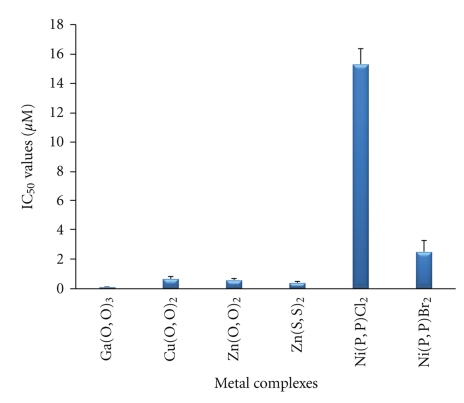
The inhibitory effect of metal complexes towards the PAF-induced WRP's aggregation.

All metal complexes under investigation inhibited the PAF-induced aggregation of WRPs. This inhibitory effect was expressed by their IC_50_ value (Figure 6). Among the metal complexes tested, Ga(O,O)_3 _ exhibited the strongest inhibitory effect towards the PAF-induced WRP's aggregation, with an IC_50_ value of 62.4 ± 45.0 nM, which is more than one order of magnitude lower compared to the IC_50_ value of the other complexes tested. The Cu(O,O)_2_, Zn(O,O)_2_ and Zn(S,S)_2_ complexes also exhibited a significant inhibitory effect, with IC_50_ values (300–600 *μ*M) being more than one order of magnitude smaller compared to the value of the Ni(P,P)Cl_2_ and Ni(P,P)Br_2_ complexes. 

**Figure 7:**
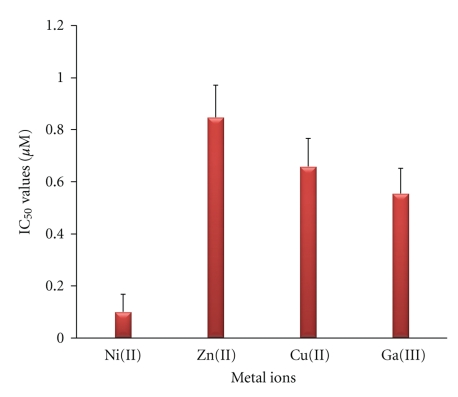
The inhibitory effect of metal salts towards the PAF-induced WRP's aggregation.

In order to assess whether the observed inhibitory effect of the metal complexes was due to their properties—mainly their three-dimensional structure—we also tested the corresponding metal salts. All metal salts examined exhibited a weak inhibitory effect, with their IC_50_ values ranging between 0.1 and 1 mM (Figure 7), significantly lower than those of the corresponding metal complexes (p < 0.001) (Figure 6). Moreover, since Ga(O,O)_3_ showed the most prominent inhibitory effect, we also examined the corresponding (OPPh_2_)(OPPh_2_)NH ligand, which has already been structurally characterized [41]. This compound exhibited a significantly smaller inhibitory effect (two orders of magnitude) against PAF (IC_50_ = 1.57 ± 0.37 *μ*M) compared with the Ga(O,O)_3_ complex (p < 0.001). This ligand's IC_50_ value is clearly smaller than those of the metal salts, but significantly greater than those of the Cu(O,O)_2_ and Zn(O,O)_2_ complexes (p < 0.05).

### 3.3. Inhibitory Effect of Metal Complexes and Metal Ions against the Thrombin-Induced WRP's Aggregation

The metal complexes exhibiting the stronger inhibitory effect against the PAF-induced WRP's aggregation were further tested for their potential inhibitory effect towards the thrombin-induced WRP's aggregation [10]. The data of Table 1 show that, among all complexes tested, Ga(O,O)_3_ did not inhibit thrombin, even at concentrations up to 10^−4^ M, while Zn(S,S)_2_ showed the strongest inhibitory effect, displaying an IC_50_ value at least one order of magnitude smaller than that of Cu(O,O)_2_ and Zn(O,O)_2_.

### 3.4. Inhibitory Effect of Metal Complexes and Metal Ions against the PAF-Induced Rabbit PRP Aggregation

**Figure 8:**
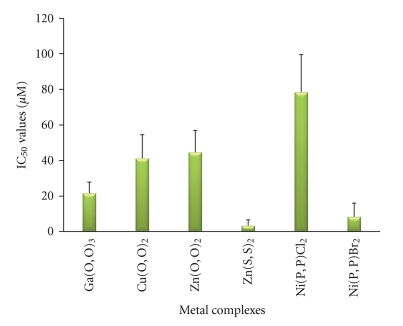
The inhibitory effect of metal complexes towards the PAF-induced rabbit PRP aggregation.

The metal complexes that inhibited the PAF-induced WRP's aggregation were further tested for their potential inhibitory effect against the PAF-induced rabbit PRP aggregation. All metal complexes indeed showed such inhibitory behavior, with IC_50_ values presented in Figure 8. The Ga(O,O)_3_, Zn(S,S)_2_ and Ni(P,P)Br_2_ complexes exhibited the highest inhibitory effect, since their IC_50_ values were significantly lower than that of all the other complexes tested (p < 0.05).

Moreover, we also tested the potential inhibitory effect of the corresponding metal salts. From all metal salts tested, only CuCl_2_ exhibited a weak inhibitory effect (IC_50_ = 12.3 ± 1.13 mM), three orders of magnitude larger than that of its corresponding Cu(O,O)_2_ complex (p < 0.001). On the contrary, the Ga(III), Ni(II) and Zn(II) salts did not inhibit the PAF-induced aggregation of rabbit PRP. Similarly, the (OPPh_2_)(OPPh_2_)NH ligand did not show such an inhibitory action.

## 4. Discussion

The successful application of several anti-PAF agents [11–24], and metal complexes [25–31] towards various inflammatory pathological situations, led us to study the effect of the metal complexes described above towards PAF-related biological activities. In that respect, we have primarily studied the *in vitro* effects of these compounds on the PAF-induced platelet aggregation. We have previously showed that *cis*-[RhL_2_Cl_2_]Cl, L = 2-(2'-pyridyl)quinoxaline, is a potent inhibitor of this type, having an IC_50_ value of 210 nM (at 0.02 nM PAF), with the inhibitory effect taking place, partly, through the PAF-receptor dependent way [42].

In this study, the biological assays were focused on the PAF-induced WRP's and rabbit PRP aggregation. In particular, our study on WRPs probes the anti-PAF activity of metal complexes under the experimental conditions applied, while, in the case of rabbit PRP, the conclusions drawn pinpoint the effect of these compounds towards the PAF activation at more similar to the *in vivo* conditions. Our work leads to the unprecedented conclusion that several metal complexes inhibited the PAF-induced aggregation towards both WRPs and rabbit PRP, in a dose-dependent manner. Significantly higher concentrations (at least one order of magnitude) of each compound were needed in order to inhibit the PAF-induced aggregation of rabbit PRP, compared to those needed in order to inhibit the corresponding aggregation of WRPs. The metal complexes with the most prominent anti-PAF activity were additionally tested towards the thrombin-induced aggregation of WRPs.

The IC_50_ values reflect the inhibition strength of each metal complex, since a low IC_50_ value reveals stronger inhibition of the PAF-induced aggregation for a given metal complex concentration. It is of significant importance that the IC_50_ values of these compounds (expressed as *μ*M) against the PAF-induced aggregation are comparable with the IC_50_ values of some of the most potent PAF receptor antagonists, namely WEB2170, BN52021, and Rupatadine (0.02, 0.03 and 0.26 *μ*M, resp.) [43–45]. This observation demonstrates that the metal complexes in question exhibit a strong inhibitory effect against the PAF activity. The octahedral Ga(O,O)_3_ complex, which contains the larger number (12) of phenyl rings in the second coordination sphere (Figure 3), is clearly the bulkier compared to the rest of the complexes studied (Figures 1, 2, and 4). This tris-chelated complex exhibited the strongest inhibitory effect against the PAF-induced aggregation of WRPs, with an IC_50_ value of 0.062 ± 0.045 *μ*M. The fact that this complex did not inhibit the thrombin-induced aggregation of WRPs, even at high doses, suggests that it antagonizes the platelet aggregation through the selective inhibition of the PAF-receptor pathway. Moreover, since the complexes of Cu(II) (square planar) and Zn(II) (tetrahedral), bearing the same (OPPh_2_)(OPPh_2_)NH ligand, exhibited an appreciable but lower inhibitory effect against the PAF-induced aggregation of both WRPs and rabbit PRP compared to Ga(O,O)_3_, it is concluded that this action depends primarily on the complexes' three-dimensional structure. This proposal is also supported by the fact that the corresponding metal salts either inhibited weakly the PAF-induced WRP's aggregation, or showed no inhibitory action at all when tested in rabbit PRP. As far as the inhibitory effect of ZnCl_2_ is concerned, our results are consistent with those reported earlier [46]. 

It should be stressed that, even though the (OPPh_2_)(OPPh_2_)NH ligand (Figure 5) inhibited the PAF-induced aggregation of WRPs, this effect was significantly less prominent compared with the effects observed in the case of the metal complexes studied. In addition, this ligand did not influence the PAF-induced aggregation of rabbit PRP, a fact that confirms the importance of specific structural characteristics of the inhibitors. Moreover, it emphasizes that the coordination of metal ions to ligands, each separately showing some PAF-inhibitory action, enhances these inhibitory properties. Similar phenomena of pharmacological profile enhancement are also reported in the case of classic anti-inflammatory drugs like indomethacin, as well as antioxidants such as bioflavonoid rutin and naringin, coordinated to transition metals [47–50]. Similar behavior was also established in the case of Cu(II) and Pt(II) complexes with various ligands [25, 50–52].

Besides their anti-PAF activity, Cu(O,O)_2_ and Zn(O,O)_2_ inhibited also the thrombin-induced aggregation of WRPs, but at higher concentrations than those tested against PAF, suggesting that these metal complexes exhibit a more general anti-inflammatory action, which, however, is more specific towards the PAF-related pathway. As far the tetrahedral Zn(S,S)_2_ complex is concerned, it exhibited the highest inhibitory effect against the PAF-induced aggregation of rabbit PRP, even when compared with that of Ga(O,O)_3_. At the same time, this complex showed also a strong inhibitory effect against the PAF-induced aggregation of WRPs, which although lower than that of Ga(O,O)_3_, it is almost twice as strong (statistically borderline significant with p = 0.065) than that of Zn(O,O)_2_ and Cu(O,O)_2_. Also, this complex inhibited strongly the thrombin-induced aggregation of WRPs, at similar concentrations with those tested against PAF in WRPs, having an IC_50_ value at least one order of magnitude lower than those of Zn(O,O)_2_ and Cu(O,O)_2_. This result suggests that Zn(S,S)_2_ exhibits a more general anti-inflammatory activity, since it can equally inhibit both the PAF and thrombin-related activities. Taking also into account that in severe inflammatory procedures implicated in cancer situations like melanoma, the PAF- and thrombin-activated pathways are interrelated, thus regulating, for instance, both the melanoma cell adhesion and its metastasis [53, 54], compounds such as Zn(S,S)_2_, Zn(O,O)_2_ and Cu(O,O)_2_, with inhibitory effects towards both PAF and thrombin-related activities, are promising candidates as potential anticancer or antithrombotic agents.

Regarding the square-planar complexes of Ni(II) studied in this work, both Ni(P,P)Cl_2_ and Ni(P,P)Br_2_ only weakly inhibited the PAF-induced aggregation of WRPs compared with the imidodiphosphinate-containing complexes. The gradual degradation of these complexes in DMSO, documented by their UV-vis spectra, is the most likely explanation for this observation. However, the fact that, in the case of rabbit PRP, the Ni(P,P)Br_2_ complex exhibited a noticeable inhibitory effect against the PAF-induced aggregation, at levels comparable to those of Ga(O,O)_3_ and Zn(S,S)_2_ (Figure 8), shows that the integrity of its structure is sufficiently retained. The observed instability of Ni(P,P)X_2_, X = Cl, Br, in DMSO renders them unsuitable for inhibitory action, at least under the conditions employed in this work. Therefore, the thrombin-induced WRP's aggregation by these complexes was not investigated.

## 5. Conclusions

A series of metal complexes, bearing dichalcogenated imidodiphosphinate ligands, inhibited the PAF-induced aggregation of both WRPs and rabbit PRP, at concentrations comparable with those of classical PAF-inhibitors. The compounds with the stronger anti-PAF activities exhibited different specificity against the thrombin-induced platelet aggregation: Ga(O,O)_3_, with the highest anti-PAF activity, did not inhibit the thrombin-related pathway, whereas Zn(S,S)_2_, with also a strong inhibitory effect against PAF, exhibited the highest inhibition against thrombin. On the other hand, Zn(O,O)_2_ and Cu(O,O)_2_ inhibited moderately both PAF and thrombin, being more effective towards PAF. The metal salts and the (OPPh_2_)(OPPh_2_)NH ligand showed no appreciable anti-PAF or antithrombin activity. By comparing the relative inhibitory activities of all compounds studied, it is concluded that the biological activities of the metal complexes depend, at least in part, on their stereochemical properties. In this respect, the more spherical and bulky structure of Ga(O,O)_3_ seems to be the most suitable for a PAF-related inhibitory action. On the contrary, this complex is totally inactive towards the thrombin-related pathway. Whether this remarkable selectivity is indeed due to a more efficient interaction of Ga(O,O)_3_ with the PAF-receptor, remains to be investigated in future studies. It is of interest that similar tris-chelated Ga(III) complexes have shown significant pharmaceutical action towards various pathological cases [27, 28]. The exploration of additional complexes of variable metal ions and three-dimensional structures is clearly needed, in an effort to further elucidate the necessary electronic or structural features for a significant and selective PAF- or thrombin-related inhibitory function.

## Figures and Tables

**Table 1:** The inhibitory effect of metal complexes towards the thrombin-induced WRP's aggregation.

Metal complex	IC_50_ value (*μ*M)
Ga(O,O)_3_	—
Cu(O,O)_2_	7.86 ± 4.77
Zn(O,O)_2_	12.80 ± 5.62
Zn(S,S)_2_	0.594 ± 0.41
